# Protective Effects of Bifidobacterium Breve MCC1274 as a Novel Therapy for Alzheimer’s Disease

**DOI:** 10.3390/nu17030558

**Published:** 2025-01-31

**Authors:** Mona Abdelhamid, Scott E. Counts, Chunyu Zhou, Hideki Hida, Jae-Il Kim, Makoto Michikawa, Cha-Gyun Jung

**Affiliations:** 1 Department of Translational Neuroscience, College of Human Medicine, Michigan State University, 400 Monroe Avenue NW, Grand Rapids, MI 49503, USA; monaabdellatif1992@yahoo.com (M.A.); scott.counts@hc.msu.edu (S.E.C.); 2 Department of Biochemistry, Nagoya City University Graduate School of Medical Sciences, Nagoya 467-8601, Japan; haruu5916@gmail.com; 3 Department of Neurophysiology and Brain Science, Nagoya City University Graduate School of Medical Sciences, Nagoya 467-8601, Japan; hhida@med.nagoya-cu.ac.jp; 4 Department of Food Science and Nutrition, Pukyong National University, Busan 48513, Republic of Korea; jikim@pknu.ac.kr; 5 Department of Geriatric Medicine, School of Life Dentistry at Niigata, Nippon Dental University, Niigata 951-8580, Japan; 6 Center for Nursing International Promotion, Nagoya City University Graduate School of Nursing, Nagoya 467-8601, Japan

**Keywords:** Alzheimer’s disease, *Bifidobacterium breve* MCC1274, amyloid-β, tau phosphorylation, glial activation, synapses

## Abstract

Alzheimer’s disease (AD) is the most common form of dementia and is characterized by memory impairment that significantly interferes with daily life. Therapeutic options for AD that substantively modify disease progression remain a critical unmet need. In this regard, the gut microbiota is crucial in maintaining human health by regulating metabolism and immune responses, and increasing evidence suggests that probiotics, particularly beneficial bacteria, can enhance memory and cognitive functions. Recent studies have highlighted the positive effects of *Bifidobacterium breve* MCC1274 (*B. breve* MCC1274) on individuals with mild cognitive impairment (MCI) and schizophrenia. Additionally, oral supplementation with B. breve MCC1274 has been shown to effectively prevent memory decline in *App^NL–G–F^* mice. In relation to Alzheimer’s pathology, oral supplementation with *B. breve* MCC1274 has been found to reduce amyloid-β (Aβ) accumulation and tau phosphorylation in both *App^NL–G–F^* and wild-type (WT) mice. It also decreases microglial activation and increases levels of synaptic proteins. In this review, we examine the beneficial effects of *B. breve* MCC1274 on AD, exploring potential mechanisms of action and how this probiotic strain may aid in preventing or treating the disease. Furthermore, we discuss the broader implications of *B. breve* MCC1274 for improving overall host health and provide insights into future research directions for this promising probiotic therapy.

## 1. Alzheimer’s Disease

Alzheimer’s disease (AD) is a progressive neurodegenerative disorder that affects the brain, causing a gradual decline in memory, thinking, and reasoning abilities. It is the most common cause of dementia, a term used to describe cognitive decline severe enough to interfere with daily life. This disease is characterized by the accumulation of abnormal protein deposits in the brain, particularly amyloid beta (Aβ) plaques and neurofibrillary tangles (NFTs). Amyloid plaques are clumps of Aβ protein that build up in the parenchyma, while intraneuronal NFTs consist of abnormally twisted tau protein fibrils and aggregates. These abnormal deposits disrupt communication between neurons, leading to excessive neuronal loss and degeneration. As a result, the brain shrinks, and microglia and reactive astrocytes become activated, contributing to blood–brain barrier (BBB) leakage and neuroinflammation, especially in areas responsible for memory and learning, such as the hippocampus [1,2,3].

The Alzheimer’s Association estimated that around 35 million individuals lived with AD globally in 2023. Due to the aging population, the global number of individuals affected by AD and other dementias could reach 152 million worldwide by 2050. The World Health Organization has emphasized the urgent need for strategies to improve early detection, prevention, and treatment of these conditions [4,5,6]. As the disease progresses, it impairs an individual’s ability to perform routine tasks, leading to increased dependence on caregivers. The exact cause of AD is not fully understood, but it is believed to result from a combination of genetic, environmental, and lifestyle factors. Despite extensive research over the past few decades aimed at understanding the origins of AD, its precise pathophysiology remains unclear, and there is currently no definitive treatment for the condition. The pathophysiology of AD involves a complex interplay of multiple factors, with hallmark features including the accumulation of Aβ plaques and NFTs in the brain, along with oxidative stress, neuroinflammation, neurodegeneration, and synaptic loss.

Aβ is produced from the cleavage of amyloid precursor protein (APP) by specific enzymes, β–secretase which first cleaves APP, followed by γ–secretase which then cleaves the remaining fragment to generate Aβ, this process is considered a key step in the development of AD [7]. In a healthy brain, Aβ is typically cleared away through enzymatic degradation, transport across the BBB, or microglial phagocytosis. However, in the brains of sporadic AD patients, this clearance process is impaired, leading to the accumulation of Aβ, which aggregates into insoluble plaques between neurons. These plaques disrupt synaptic function and contribute to neuronal toxicity through several mechanisms, including oxidative stress, inflammation, and calcium dysregulation. Ultimately, this leads to impaired neuronal communication and promotes cell death and disconnection [8,9].

The NFTs are composed of hyperphosphorylated tau protein. Under normal conditions, tau stabilizes microtubules, which are essential for maintaining the structure of neurons and facilitating the transport of nutrients and signaling molecules. In AD, tau undergoes abnormal hyperphosphorylation that causes tau to detach from microtubules and aggregate into twisted, insoluble tangles. The loss of tau’s ability to stabilize microtubules disrupts intracellular transport, impairs neuronal function, and ultimately leads to neuronal degeneration and death [10].

Microglial activation occurs in response to the accumulation of Aβ plaques and NFTs, leading to the release of pro-inflammatory cytokines and triggering a neuroinflammatory response. While this activation serves as a natural immune defense, chronic inflammation exacerbates neuronal damage and accelerates disease progression [11]. Key cytokines, such as tumor necrosis factor–alpha (TNF–α) and interleukin–1 beta (IL–1β), contribute to synaptic dysfunction, disrupting communication between neurons and impairing cognitive functions like memory [11]. As the disease progresses, synaptic loss and neuronal death become more widespread, particularly in regions critical for memory, such as the hippocampus and cortex. This ultimately leads to brain atrophy and progressive cognitive decline [8,12].

Finding an effective treatment for AD is crucial not only to alleviate suffering and improve the quality of life for millions of patients but also to reduce the significant societal, economic, and emotional costs associated with the disease. Currently, there are a few FDA-approved drugs, such as acetylcholinesterase inhibitors (e.g., donepezil and rivastigmine) and glutamate regulators (memantine). However, these medications provide only limited symptomatic relief and do not halt or reverse the underlying progression of the disease [13,14]. These treatments primarily target neurotransmitter imbalances but fail to address the root causes of AD, such as the accumulation of Aβ plaques and abnormal tau proteins. Additionally, these medications do not improve memory in patients with mild cognitive impairment (MCI) or early-stage AD and can cause several adverse effects, including nausea, vomiting, hallucinations, confusion, dizziness, headaches, and fatigue [15,16]. Therefore, there is an urgent need to develop an effective treatment for AD that addresses cognitive deficits, anxiety, depression, and other behavioral issues.

Recent research has highlighted the significant role of gut microbiota in modulating brain function and its potential involvement in the pathophysiology of AD. The gut–brain axis, a bidirectional communication pathway between the central nervous system (CNS) and the gut, has been increasingly recognized as a critical factor in the neuroinflammation and neurodegeneration observed in AD. Imbalances in the gut microbiota, known as dysbiosis, have been linked to heightened neuroinflammatory responses and cognitive decline. Given the emerging evidence supporting the influence of gut health on brain function, probiotics, beneficial bacteria that can help restore microbiome balance, are being explored as a promising therapeutic avenue to mitigate AD-related pathology. This shift in focus towards microbiome modulation offers a novel perspective on the prevention and treatment of AD, complementing traditional approaches.

## 2. Role of Gut Microbiota in Neurodegenerative Diseases

In recent decades, research has increasingly focused on the potential role of gut microbiota, trillions of microorganisms residing in the gastrointestinal tract (GIT), in the development and progression of neurodegenerative diseases. These studies emphasize the complex bidirectional communication between gut microbiota and the brain through the gut–brain axis. This interaction suggests that gut microbiota can influence cognitive and brain functions, as well as neuroinflammation. The gut microbiota plays a crucial role in regulating the immune system; dysbiosis can lead to chronic low-grade inflammation, which may trigger neuroinflammatory responses that affect the brain [17]. Microglia, the brain’s innate immune cells, are particularly influenced by gut-derived inflammatory signals. Overactive microglia can worsen conditions like AD by releasing pro-inflammatory cytokines that damage neurons and synapses [18,19,20].

Additionally, gut microbiota produces neuroprotective short–chain fatty acids (SCFAs), such as butyrate, acetate, and propionate, which can cross the BBB and possess anti-inflammatory properties [21,22]. Notably, butyrate supports neuronal health by promoting neurogenesis and synaptic plasticity along with reducing oxidative stress [23,24]. Recent studies suggest that gut microbiota may significantly influence the accumulation and clearance of Aβ in the brain. Certain gut bacteria, like *Bacteroides fragilis*, may promote Aβ accumulation by inhibiting its clearance by microglia, leading to plaque deposition in AD models [25]. Conversely, some research indicates that probiotics, especially *bifidobacteria* and lactic acid bacteria, can reduce inflammatory markers and Aβ deposition in the brain [26]. Patients with AD often have a lower proportion of butyrate–producing bacteria, which are known to reduce inflammation, and a higher proportion of bacteria that promote inflammation [27]. Furthermore, gut microbiota can affect the activity and expression of Aβ–degrading enzymes, such as neprilysin and insulin–degrading enzyme (IDE). Healthy microbiota, through probiotic treatments or dietary interventions, may enhance neprilysin’s expression by SCFA production [28]. Experiments with germ-free mice have shown increased levels of Aβ-degrading enzymes compared to conventionally raised mice, suggesting that gut microbiota can influence Aβ levels [29].

Additionally, gut microbiota significantly impacts the permeability of the BBB, which protects the brain from harmful substances. A disrupted BBB is a critical factor in neurodegenerative diseases like AD [30]. A healthy microbiota, along with the recolonization of germ-free mice with microbiota from pathogen-free mice, can promote BBB integrity through butyrate production, which stimulates mucin secretion to prevent lipopolysaccharide (LPS) absorption and suppress inflammation [22,31]. In contrast, dysbiosis can increase BBB permeability by reducing the expression of tight junction proteins, such as occludin and claudin-5, facilitating the entry of toxins and inflammatory mediators into the brain [32,33,34]. Overall, the relationship between gut microbiota and the BBB presents potential avenues for intervention in managing AD and other neurodegenerative disorders.

The enteric nervous system (ENS), often referred to as the second brain, is a complex network of neurons located within the GIT that regulates various digestive processes. Recent evidence indicates that gut microbiota significantly impacts ENS function; disruptions in microbial balance can lead to gastrointestinal disturbances linked to neurological disorders [35,36]. In Parkinson’s disease (PD), ENS dysfunction, which begins in the gut and may extend to the brain, can contribute to the development of motor symptoms [35], possibly triggered by ENS accrual of α-synuclein, which may be transported by the vagus nerve to seed Lewy body pathology in the brain, influencing disease progression [37].

Research suggests that gut microbiota plays a significant role in influencing behavior and cognitive function by altering the levels of neurotransmitters, particularly serotonin and dopamine [38]. These neurotransmitters are crucial for mood regulation and cognitive processes. Additionally, the metabolites produced by gut microbiota may impact brain activity and directly affect regions associated with cognition, such as the hippocampus [28,39]. Modulating gut microbiota through dietary interventions, prebiotics, probiotics, or fecal microbiota transplantation has been proposed as a potential therapeutic strategy for neurodegenerative diseases [40]. For instance, changing the composition of gut microbiota may help reduce inflammation, improve cognitive function, and enhance overall brain health [41,42,43,44,45]. Currently, clinical trials are underway to explore the potential of microbiota-based therapies for treating neurodegenerative diseases. However, further research is needed to understand the exact mechanisms involved.

## 3. Gut–Brain Axis

The gut–brain axis refers to the bidirectional communication between the brain and GIT, facilitating continuous signaling that influences various physiological functions, including mood, cognition, immune regulation, and behavior. The gut microbiota plays a crucial role in modulating this complex and dynamic pathway. Key mechanisms of the gut–brain axis involve neural, immune, endocrine, and hormonal connections that enable communication between the brain and gut, along with the influence of microbes. The vagus nerve is the primary pathway for this communication, serving as a vital link between the gut and the brain. Signals from the gut, such as those generated by mechanical stretching or microbial activity, are transmitted to the brain through the vagus nerve. This pathway affects various brain functions, including emotional regulation and stress responses [46]. Consequently, the brain can send signals to the gut to help regulate digestion, while the gut can inform the brain about its condition, such as feelings of fullness or discomfort, through the sensory fibers of the vagus nerve.

Moreover, the gut microbiota influences the immune system by regulating systemic inflammation, which in turn affects brain function. Chronic low-grade inflammation, characterized by continuous production of pro–inflammatory cytokines like TNFα, IL–1, IL–6, and interferon-gamma (IFNγ), is associated with neuroinflammation, a key feature of neurodegenerative diseases [47]. SCFAs inhibit histone deacetylases (HDACs) [48], which can reduce inflammation in macrophages and dendritic cells [49], activate G protein-coupled receptors, promote the development of regulatory T cells (Tregs), and increase the production of antibacterial effectors that help macrophages eliminate bacteria [48,50,51]. Additionally, butyrate has been shown to exert anti-inflammatory effects and promote neurogenesis [52].

The gut microbiota also influences the hypothalamic–pituitary–adrenal (HPA) axis, which is responsible for the body’s stress response. Dysbiosis, or microbial imbalance, can disrupt HPA axis activity, contributing to stress-related disorders such as depression and anxiety [53]. The gut microbiota significantly influences brain function and behavior, as it is essential for producing monoamine neurotransmitters such as serotonin, dopamine, and norepinephrine, as well as GABA, all of which directly affect mood, anxiety, and cognitive function. Notably, approximately 90% of the body’s serotonin, a key neurotransmitter involved in mood regulation, is synthesized in the gut. Dysbiosis has been linked to various neurological and psychiatric disorders, including depression, anxiety, and cognitive decline [54,55]. As a result, probiotics, prebiotics, and dietary interventions that modify the gut microbiota are emerging as important therapeutic strategies for enhancing brain health. For instance, supplementation with probiotics such as *Lactobacillus* and *Bifidobacterium* has shown promise in improving mood, cognitive function, and resilience to stress in both animal models and human studies [56]. These probiotics may enhance the production of SCFAs, which benefit brain health by reducing inflammation and supporting neurogenesis.

Overall, the gut–brain axis is a complex system that highlights the significant impact of gut health on brain function and mental well–being. Understanding how the gut microbiota affects neurological health opens up exciting possibilities for new treatments for conditions like depression, anxiety, and neurodegenerative diseases. Probiotics, prebiotics, and dietary modifications present promising non-pharmacological interventions for improving both gut and brain health, particularly as research continues to explore the role of the gut–brain axis in cognitive function and behavior.

## 4. *Bifidobacterium breve* MCC1274 as a Multifaceted Probiotic for the Gut and Brain

### 4.1. Characterization of Bifidobacterium breve MCC1274 and Its Benefits on Gut Integrity

*Bifidobacterium breve* (*B. breve*) MCC1274 is a specific strain of the *Bifidobacterium* genus that is commonly found in the human microbiota, particularly in the gut microbiome of infants [57,58]. Like other species within the *Bifidobacterium* family, *B. breve* MCC1274 is a Gram-positive, anaerobic, rod-shaped, non-motile, and non-spore-forming bacterium. It typically exhibits a Y–shaped or branched structure [59,60]. This strain is known for its ability to ferment carbohydrates, particularly oligosaccharides and dietary fibers, resulting in the production of lactic acid and acetic acid. These metabolites help lower the pH of the gut, creating an environment that is unfavorable for pathogenic bacteria [60,61]. *B. breve* MCC1274 commonly referred to as “*B. breve* A1”, is generally considered safe and has shown good tolerability. It has been studied for its specific probiotic benefits, particularly with cognitive function and gut health. Research suggests it may have potential benefits for individuals with MCI due to its unique ability to protect against brain atrophy and enhance memory function [62]. This effect may be related to its capacity to modulate the gut–brain axis through neurotransmitter production and interactions with the immune system [38,63].

*B. breve* is a component of the natural gut microbiota, especially in the intestines of breastfed infants. It is predominantly found in the large intestine, where it plays a crucial role in maintaining a healthy gut ecosystem. In addition to being present in the intestines, *B. breve* can also be isolated from other environments, such as fermented foods and animal intestines [64,65,66,67]. This bacterium is essential for maintaining the intestinal barrier and supporting the integrity of the intestinal epithelial lining. This function is crucial for preventing harmful pathogens and toxins from entering the bloodstream, potentially leading to inflammation and contributing to diseases such as inflammatory bowel disease (IBD) [68,69]. Moreover, *B. breve* enhances tight junction proteins by increasing the expression of key proteins like occludin and zonula occludens, which help strengthen the gut lining [70]. Furthermore, *B. breve* modulates immune responses in the gut by interacting with gut-associated lymphoid tissue (GALT). It promotes the production of anti-inflammatory cytokines, such as IL–10 and transforming growth factor–beta 1 (TGF–β1), which help regulate immune tolerance and reduce inflammation [71]. It has been indicated that *B. breve* may alleviate conditions causing inflammation, such as colitis, thus assisting in managing issues related to immune system imbalances [68].

Additionally, it contributes to a healthy gut microbiota by promoting the growth of beneficial bacteria, including other *bifidobacteria* and *lactobacilli*, leading to a more balanced microbial ecosystem [69]. *B. breve* produces lactic acid and other antimicrobial substances that can inhibit harmful pathogens in the gut, like *Escherichia coli* and *Clostridium difficile*. By fermenting oligosaccharides and fibers, *B. breve* may provide prebiotic effects that nourish beneficial bacteria and further support overall gut health [72]. Therefore, *B*. *breve* is a probiotic strain that supports gut health through multiple mechanisms, maintains gut barriers, supports immune function by preventing inflammation, and helps maintain a healthy balance of gut microbiota. These properties make it beneficial for improving overall gut health, preventing gastrointestinal disorders, and promoting immune system regulation.

### 4.2. Probiotic Properties of B. breve MCC1274

*B. breve* is thought to influence the gut–brain axis, impacting neuroinflammation, oxidative stress, and cognitive function. This strain can modulate neuroinflammation by affecting the gut–brain axis, potentially reducing inflammation in the brain by influencing pro-inflammatory cytokines associated with neurodegenerative diseases and reducing immune-reactive genes [73,74,75]. It balances pro-inflammatory and anti–inflammatory cytokines, contributing to a more favorable environment for brain health. Moreover, *B. breve* may help reduce oxidative stress through its metabolic products or by increasing the activities of antioxidant enzymes, protecting neural cells from oxidative damage [76,77,78]. Administration of *B. breve* in animal models has been linked to improved cognitive function, including enhanced memory and learning abilities, likely due to its anti-inflammatory and antioxidant properties [74,76].

This strain has also been associated with improved cognitive performance in aging and better learning and memory in preclinical studies (Table 1 and Table 2). Notably, *B. breve* has led to significant improvements in cognitive function in individuals with MCI, likely linked to its ability to modulate the gut–brain axis and influence neurotransmitter production, as well as its potential effects on neuroinflammation and oxidative stress [62,79,80,81]. Moreover, supplementation with *B. breve* MCC1274 has been shown to result in significant reductions in both anxiety and depressive symptoms in patients with schizophrenia. Notably, this probiotic was well tolerated by participants, with no major adverse effects reported during the study period [82]. Overall, these findings underscore the potential of *B. breve* MCC1274 as a promising therapeutic option for the management of neurodegenerative dementing disorders, further supporting its potential role in brain health.

## 5. Mechanisms of Action of *B. breve* MCC1274 in Neurodegenerative Disease

### 5.1. Potential Effects of B. breve MCC1274 Supplementation in Modulating Neuroinflammation

CNS innate immune dysfunction plays a crucial role in the development of AD and can begin decades before patients exhibit any signs of cognitive impairment. This process involves the activation of glial cells, which are closely associated with the accumulation of Aβ and tau proteins in the brain. The activation of glial cells leads to the release of pro-inflammatory cytokines, creating an imbalance between pro-inflammatory and anti-inflammatory responses, which heightens the sensitivity of the brain to inflammatory factors [86,87,88]. *B. breve* MCC1274 is currently recognized as a promising probiotic bacterium that influences the gut–brain axis by modulating the immune system (Table 1 and Table 2) and could play a role in slowing the progression of neurodegenerative diseases and cognitive decline associated with excessive brain inflammation.

It has been shown that intracerebroventricular injection of Aβ into the brains of ddy mice significantly upregulated gene expression linked to immune responses and reactions to external stimuli in the hippocampus. Surprisingly, nearly all differentially expressed genes observed in Aβ-injected mice returned to normal expression levels in the hippocampus of mice administered *B. breve* MCC1274. This finding indicated that this probiotic effectively suppresses the toxicity induced by Aβ and normalizes the gene expression profile [75]. Additionally, research has demonstrated that supplementation with *B. breve* MCC1274 reduces the production of pro-inflammatory cytokines, such as IL–1β and TNF–α, which are associated with neuroinflammation. *B. breve* MCC1274 supplementation significantly decreased both the level of the Iba1 protein and the number of Iba1^+^ cells surrounding Aβ plaques in the hippocampus of *App^NL-G-F^* mice. Furthermore, *B. breve* MCC1274 supplementation increased the expression of anti-inflammatory cytokines such as TGF–β1 while decreasing the mRNA expression of pro-inflammatory cytokines IL–6 and IL–1β in the hippocampus of *App^NL–G–F^* mice [74]. This suggests that this probiotic may influence microglial behavior by promoting a shift from a pro-inflammatory M1 phenotype to an anti-inflammatory M2 phenotype.

Moreover, *B. breve* can influence the microbiome and promote the production of certain metabolites by increasing plasma levels of genistein in *App^NL–G–F^* and wild-type (WT) mice, as well as acetate levels in Aβ–injected mice. These increases may enhance anti–inflammatory pathways, support microglial polarization toward an anti–inflammatory phenotype, and provide metabolic support to neurons [75,78]. Moreover, *B. breve* MCC1274 supplementation has been found to attenuate microglial activation in WT mice by activating the protein kinase B (Akt)/glycogen synthase kinase–3β (GSK–3β) pathway, significantly reducing the number of Iba1^+^ cells in the hippocampus. Enhanced Akt activation in the brains of mice supplemented with *B. breve* MCC1274 may, therefore, play a crucial role in reducing microglial activation [73]. Furthermore, *B. breve* MCC1274 may mitigate inflammation by limiting the formation of lipid droplets in vitro. It significantly reduces the expression of perilipin 4, a protein associated with lipid droplet formation that is known to increase during inflammatory conditions in SH–SY5Y cells [77]. Overall, *B. breve* MCC1274 is being explored as a potential therapeutic strategy for neurodegenerative conditions like AD, where chronic inflammation plays a significant role.

The gut microbiota also plays a crucial role in regulating lipid and energy metabolism, which significantly impacts overall health, including mental well–being. One major way the gut microbiota influences health is through the production of SCFAs. These SCFAs help maintain a healthy gut environment by promoting a balanced microbial community and reducing systemic inflammation [89]. Chronic inflammation is often linked to various mental health disorders, such as depression and anxiety. Therefore, a well–regulated gut microbiota can help reduce inflammation and support mental health. *B. breve* has been shown to influence lipid metabolism in patients with schizophrenia by promoting the production of SCFAs [90], which supports gut health and encourages the growth of beneficial microbes that may have anti–inflammatory effects. In this way, *Bifidobacterium* helps balance the gut microbiota, potentially reducing inflammation and fostering a healthy metabolic environment. Additionally, *B. breve* MCC1274 showed potential anti–inflammatory properties in subjects with MCI, possibly linked to its metabolite acetate, which may contribute to observed cognitive improvements. This probiotic could modulate the gut microbiota by reducing pro–inflammatory bacteria and promoting intestinal barrier integrity. Furthermore, *B. breve* MCC1274 produces anti–inflammatory metabolites, such as indole–3–lactic acid, which could influence brain inflammation and cognitive function [81,91,92,93].

### 5.2. Modulation of Oxidative Stress and Chronic Stress Responses by B. breve MCC1274 Supplementation

Oxidative stress significantly contributes to the pathology of AD by damaging neurons and other brain cells. This stress is often linked to the accumulation of Aβ plaques, which exacerbate the inflammatory response and neuronal degeneration. In AD, there is an excess production of reactive oxygen species (ROS), leading to cellular damage, inflammation, and activation of microglia., which in turn creates a cycle of neuroinflammation and neuronal dysfunction [94,95].

Supplementation with *B. breve* MCC1274 has been shown to increase plasma levels of 5-methoxyindoleacetic acid in addition to genistein, both of which exhibit potential antioxidative activity (Table 3). These metabolites may work systemically to reduce oxidative stress not only in the brain but throughout the body, providing broader protective effects in *App^NL–G–F^* mice. The increased levels of antioxidative metabolites could also influence other pathways related to inflammation and cellular repair, contributing to overall brain health. These metabolites can neutralize ROS, thereby diminishing oxidative stress in the brain and protecting neurons and glial cells from oxidative damage. This antioxidative effect may help to slow down or prevent the neurodegenerative processes associated with AD in this AD mouse model [78].

Furthermore, *B. breve* MCC1274 plays a vital role in protecting neurons from the accumulation of lipid droplets. This probiotic influences the expression of the perilipin 4 gene, a key regulator of lipid droplets in cells. By modulating the expression of perilipin 4, *B. breve* MCC1274 helps to protect neuronal cells from lipid-related damage and reduces the buildup of lipid droplets, processes often linked to metabolic dysfunction and neuronal stress [77].

**Figure 1:**
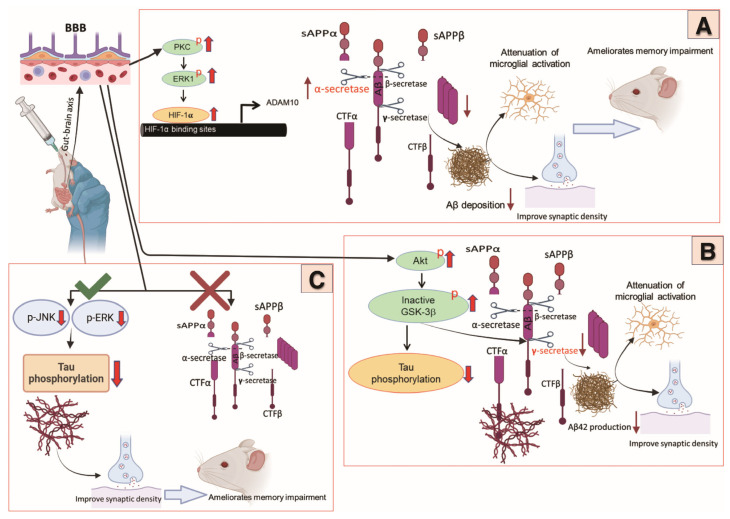
Summary of the effect of B. breve MCC1274 on *App^NL–G–F^* and WT Mice. (**A**) In 6-month-old *App^NL–G–F^* mice, oral supplementation with *B. breve* MCC1274 alleviated cognitive dysfunction and reduced Aβ deposition by increasing the ADAM10 protein level in the hippocampus. This treatment also helped alleviate neuroinflammation and improved synaptic density. (**B**) In WT mice, supplementation with *B. breve* MCC1274 decreased levels of soluble Aβ42 and PS1 proteins through the Akt/GSK–3β signaling pathway. It also attenuated microglial activation and inhibited tau phosphorylation, which may enhance synaptic function. (**C**) In 17–month–old *App^NL–G–F^* mice, administration of *B. breve* MCC1274 may provide ameliorative effects by reducing chronic stress. This is achieved by inhibiting tau hyperphosphorylation through the p–JNK and p–ERK pathways, as well as improving synaptic density.

Chronic stress is a well-established risk factor for AD and other neurodegenerative conditions. It tends to exacerbate the pathological features of AD, including the accumulation of Aβ plaques and the activation of microglia. Chronic stress adversely affects brain function through the sustained release of stress hormones like cortisol, leading to neuroinflammation and oxidative stress. In AD, chronic stress can worsen cognitive decline by promoting the formation of amyloid plaques and triggering additional inflammatory responses, thereby accelerating disease progression [96,97,98]. *B. breve* MCC1274 supplementation has a modulatory effect on stress-related responses in the *App^NL–G–F^* mice. By influencing the gut–brain axis, this probiotic may help regulate stress-related pathways, including those linked to the HPA axis. The HPA axis is a central component of the body’s stress response, and its chronic activation can result in persistently high cortisol levels, negatively impacting brain regions involved in memory and cognition, such as the hippocampus [99,100,101,102]. By positively influencing the microbiome and gut health, *B. breve* MCC1274 may help balance the HPA axis, thereby mitigating the effects of chronic stress on the brain. It has been found to effectively lower chronic stress levels in the brains of AD model mice. This effect may occur by helping to restore the balance between ROS and antioxidant defenses, which is often disrupted in AD and contributes to disease progression through cellular damage [94,103,104]. Oral administration of *B. breve* MCC1274 resulted in decreased levels of chronic stress markers, including phosphorylated JNK, ERK1/2, and HSP90, in the hippocampus and cortex of AD model mice (Figure 1). This indicates that the probiotic effectively reduces stress-related cellular responses in the brain, which is crucial for protecting neurons. By reducing chronic stress, *B. breve* MCC1274 is essential for upregulating the c–Fos protein, suggesting that this probiotic treatment enhances neuronal activity in these brain areas. This increase in activity is vital for alleviating the neuronal dysfunction and degeneration associated with AD [76].

### 5.3. Potential Effects of B. breve MCC1274 Supplementation in Modulating Aβ and Tau Pathology

Aβ is a small protein produced from APP processing by certain enzymes. Normally, APP is involved in cellular processes including synaptic regulation, but aberrant APP processing of Aβ and reduced Aβ clearance can result in extracellular aggregates of pathologic Aβ plaques. In AD, Aβ plaques disrupt neuron communication and promote inflammation. These plaques contribute to neurotoxicity, leading to impaired synaptic function, neuronal cell death, and cognitive decline. Plaques are mainly found in limbic and higher-order brain areas involved in memory, such as the hippocampus and cortex. The amyloid hypothesis suggests that Aβ plaques drive AD by triggering inflammation and causing tau protein tangles. However, some researchers argue that Aβ may be a consequence of the disease rather than its cause [76,105,106,107,108]. Treatment strategies targeting Aβ include monoclonal antibodies (e.g., ecanemab, donanemab) and enzyme inhibitors to reduce plaques. However, these treatments have had mixed results, with some showing limited effectiveness or side effects like brain swelling [83,109,110,111]. Overall, Aβ is a key factor in AD, but other factors like tau tangles and genetic predispositions also contribute to disease progression.

*B. breve* MCC1274 is believed to reduce the aggregation of Aβ or promote its clearance through both direct and indirect pathways (Table 1). One potential mechanism by which *B. breve* MCC1274 may influence AD pathology is through the gut–brain axis. This pathway suggests that gut microbiota can affect brain function and inflammation, which may in turn impact AD progression. This probiotic strain has been observed to reduce neuroinflammation by modulating microglial activation, which can contribute to Aβ accumulation by creating an environment conducive to aggregation [73,74,77,112]. Additionally, *B. breve* MCC1274 may modulate the excessive immune response triggered by Aβ injection by altering gene expression in the hippocampus of ddy mice, thereby alleviating Aβ–induced toxicity [75]. Furthermore, *B. breve* supports gut health, promoting a healthy gut microbiota that helps maintain the integrity of the BBB, facilitating the clearance of Aβ from the brain [113]. This may help mitigate the neurotoxic effects of Aβ. It also has shown that *B. breve*-mediated production of beneficial SCFAs such as acetate can decrease amyloid pathology by lowering the levels of both soluble and insoluble fractions of hippocampal Aβ 1–42 [75,78,114].

Several studies have investigated the direct effects of *B. breve* MCC1274 on the aggregation and production of Aβ (Figure 1). It has been indicated that supplementation with *B. breve* MCC1274 significantly elevates levels of ADAM10 (a disintegrin and metalloprotease 10), an enzyme crucial for cleaving Aβ in the brain. This increase occurs in the hippocampus of 7–month–old *App^NL–G–F^* mice, leading to enhanced cleavage of APP into non-toxic soluble Aβ fragments and subsequent reduction of potentially aggregating Aβ species such as Aβ42 [74]. In support of this, *B. breve* MCC1274 was shown to lower the levels of Aβ42 in the hippocampus of WT mice. *B. breve* MCC1274 may achieve this reduction by downregulating the expression of presenilin 1 (PS1), a key protein involved in the processing of APP that leads to Aβ production [73]. However, it was observed that supplementation with *B. breve* MCC1274 did not significantly reduce Aβ levels in the brains of 17–month–old *App^NL–G–F^* mice, despite potential improvements in cognitive function [76]. This lack of an effect on Aβ levels in aged *App^NL–G–F^* mice could be attributed to the advanced stage of amyloid pathology in these animals, which could reduce the efficacy of Aβ–reducing interventions. While the potential of *B. breve* MCC1274 to mitigate Aβ aggregation and toxicity in AD models is promising, further research is needed to fully understand the underlying mechanisms and to translate these findings into human applications.

Tau hyperphosphorylation plays a critical role in AD, driving neurodegeneration through the formation of NFTs [115,116]. While Aβ has long been the primary focus of research, tau pathology is equally important in the progression of cognitive decline and memory loss in AD. Recent studies have shifted attention toward targeting abnormally hyperphosphorylated tau as a potential therapeutic strategy for AD [117,118,119]. Under normal conditions, tau stabilizes microtubules, regulates their assembly, and supports neuronal growth and structure. In AD, however, tau becomes abnormally phosphorylated, impairing its ability to bind to microtubules. This destabilization disrupts the neuronal transport system, interfering with communication between neurons, and resulting in the formation of filamentous tau tangles (NFTs), a hallmark AD pathology. The accumulation of NFTs contributes to neuronal dysfunction, cell death, and the cognitive decline and memory loss characteristic of the disease [120,121]. Ongoing research seeks to better understand the role of tau in AD and develop targeted therapies that address tau-related processes.

It has been shown that supplementing with *B. breve* MCC1274 can reduce the levels of phosphorylated tau protein at specific sites, such as Thr231 (AT–180) and Ser202/Thr205 (AT–8), in aged *App^NL–G–F^* mice [76]. This reduction occurs without significantly altering total tau levels, primarily in the hippocampus. Another study demonstrated that administering *B. breve* MCC1274 to WT mice also resulted in a significant reduction of phosphorylated tau protein at the same sites (Thr231, Ser202, and Thr205) in the hippocampus [73]. This reduction is thought to be mediated through the regulation of signaling pathways like the Akt/GSK–3β pathway, which plays a crucial role in tau phosphorylation [122,123,124]. Due to its ability to modulate tau pathology in both aged *App^NL–G–F^* and WT mice, *B. breve* MCC1274 is considered a potential therapeutic target for AD; however, further research is needed to confirm its efficacy in humans. It is worth noting that another study investigated the effect of this probiotic on tau phosphorylation in young *App^NL–G–F^* mice and found that *B. breve* MCC1274 supplementation did not alter tau phosphorylation at either AT180 or PHF1 (Ser396/Ser404) epitopes in the cortex and hippocampus, nor did it affect total tau protein levels [74] (Figure 1).

### 5.4. Modulation of Gut Microbiota and Metabolic Function by breve MCC1274 Supplementation

*B. breve* MCC1274 has been shown to positively affect gut microbiota by potentially enhancing the population of beneficial bacteria, producing advantageous metabolites like SCFAs, and modulating immune responses, all while not significantly altering the overall composition of the gut microbiota, according to recent research [74,75] (Table 3). Studies indicate that supplementing with *B. breve* MCC1274 does not drastically change the overall gut microbiota, meaning it does not significantly increase or decrease the abundance of other bacterial species. Instead, it primarily influences gut microbiota through the production of metabolites such as indole–3–lactic acid (ILA), which can impact intestinal barrier function and immune regulation, rather than causing substantial changes to bacterial diversity itself [125]. 

Furthermore, antibiotic treatments can reduce microbial diversity and alter gut microbiota. In contrast, oral administration of *B. breve* MCC1274 did not significantly restore this diversity but did increase the relative abundance of *Actinobacteria* and the total counts of *B. breve* in female Wistar rats [113]. While probiotics typically elevate the numbers of specific bacteria, they rarely change the overall gut composition in experimental animals. Instead, probiotic interventions may influence the metabolic activities of gut microbes. Specifically, *B. breve* MCC1274 was found to enhance the bioavailability of daidzein by increasing its total plasma levels in rats that had been pre-treated with antibiotics [113]. This suggests that *B. breve* can hydrolyze daidzin into a more absorbable form, daidzein. Additionally, *B. breve* MCC1274 stimulated an increase in beneficial gut commensals, particularly *Actinobacteria*, which are vital for maintaining gut health. A high abundance of *Bifidobacterium*, a subgroup of *Actinobacteria*, is associated with improved gut metabolic activities [113]. Overall, *B. breve* MCC1274 may play a role in modulating gut microbiota and enhancing metabolic functions related to daidzin. Further research is necessary to explore its interactions with gut microbiota, suggesting that *B. breve* MCC1274 could serve as a novel probiotic to improve daidzein absorption and potentially alleviate health issues in humans.

Supporting the findings of the limited effect of this probiotic on gut microbiota, no significant differences were observed in the composition of gut microbiota at the phylum level in AD-like mice. However, some minor changes occurred following probiotic administration; the proportions of the phylum *Actinobacteria* and family *Bifidobacteriaceae* were significantly higher, while the families *Odoribacteraceae* and *Lachnospiraceae* were slightly lower after *B. breve* MCC1274 supplementation in AD–like mice [75]. Consistent with this finding, no marked change in gut microbiota was observed after the administration of *B. breve* MCC1274 in *App^NL–G–F^* mice. This suggests that mechanisms other than changes in gut microbial composition may contribute to the beneficial effects of this probiotic in alleviating AD pathologies in these mice [74]. As shown in earlier research, both viable and nonviable *B. breve* MCC1274 could suppress the expression of inflammatory genes in the hippocampus and improve cognitive impairment in AD–like mice, highlighting that certain structural components of the probiotics may play a role in modulating neuronal immune responses. Therefore, one possible mechanism by which probiotics help ameliorate AD–like pathologies is through these structural components. However, further investigation is needed to determine whether nonviable *B. breve* MCC1274 has beneficial effects on AD–like conditions.

In patients with schizophrenia, the composition of gut microbiota did not significantly change after four weeks of daily consumption of *B. breve* MCC1274 [82]. However, this probiotic did improve anxiety and depressive symptoms in these patients. It has been suggested that there is no direct link between changes in gut microbiota and the effects of *B. breve* MCC274 on anxiety and depression. They attributed the beneficial effects of the probiotic to the enhancement of gut epithelial barrier function, which is caused by the upregulation of interleukin (IL)–22 and tumor necrosis factor–related activation–induced cytokine (TRANCE) in responding patients [82]. These compounds are known to play important roles in gut health [126,127].

Additionally, individuals with MCI have been observed to have decreased levels of *Bifidobacterium* and increased levels of *Prevotella*, *Clostridiaceae*, *Ruminococcaceae*, and *Phascolarctobacterium* [128]. While *Prevotella* is linked to gut inflammation, *Phascolarctobacterium* is known to produce beneficial SCFAs [129,130]. This study reports for the first time that differences in gut microbiota among MCI subjects are related to the severity of cognitive impairment. Over a 24–week study, fluctuations in microbiota were noted in the placebo group, while the probiotic group did not show significant changes. This suggests that this probiotic may help maintain gut balance, which can, in turn, influence cognitive health, but its effects on altering gut microbiota may be limited [80]. Another study involving MCI subjects observed an improvement in memory functions, indicating that this enhancement might result from a shift in gut microbiota towards less pro-inflammatory bacterial species [81]. Many of these species are known to release LPS and other metabolites that can lead to microglial activation in the brain [131]. However, this study did not compare the changes in gut microbiota after treatment. Although the findings are promising, more research is needed to fully understand the exact mechanisms by which *B. breve MCC1274* impacts the gut microbiota and its subsequent effects on mental health.

### 5.5. The Impacts of B. breve MCC1274 Supplementation on BBB Integrity

In AD, a dysfunctional BBB significantly accelerates disease progression by enabling the accumulation of toxic proteins like Aβ, triggering inflammatory responses, hindering the natural waste clearance mechanisms in the brain, and dysregulating cerebral hemodynamics, ultimately contributing to neuronal damage and cognitive decline [132,133]. Restoring BBB integrity and improving its function is an area of active research, intending to slow or halt AD progression. Some research indicates that *B. breve* MCC1274 may positively impact brain health by reducing inflammation and improving cognitive function. However, there is currently no direct evidence suggesting that it significantly alters the integrity of the BBB. Ongoing studies are exploring its effects on the gut–brain axis and potential mechanisms related to brain protection, which could clarify its influence on the BBB. However, since *B. breve* MCC1274 modulates metabolites in the gut–brain axis that reduces inflammation, microglial activation, and Aβ accumulation [73,74,75], this probiotic might indirectly protect the BBB [134,135,136]. Moreover, since *B. breve* MCC1274 also has anti-inflammatory effects in the periphery, it may help mitigate systemic inflammation that can impact the brain and its BBB (Table 1). More targeted investigations are needed to determine whether *B. breve* MCC1274 can directly protect or restore BBB integrity.

### 5.6. The Effects of B. breve MCC1274 Supplementation on Cellular Proliferation and Neuronal Cell Loss

Adult hippocampal neurogenesis occurs throughout adulthood and contributes to cognitive functions and this process was found to be significantly reduced very early in AD patients compared to age-matched healthy controls [137]. In addition, cell proliferation plays a critical role in Alzheimer’s research, shedding light on the abnormal re-entry into the cell cycle that occurs in affected neurons. This re-entry is considered a key mechanism contributing to neuronal death in the disease. Unlike healthy adult neurons, which typically do not divide, neurons in AD show signs of re-entering the cell cycle but fail to complete it properly, leading to cellular dysfunction and programmed cell death [138,139].

Our previous studies indicate that *B. breve* MCC1274 does not significantly affect neurogenesis in the hippocampus (Table 1). We found no significant increase in BrdU–positive cells in the subgranular zone of the dentate gyrus, a region where new neurons are usually generated in both *App^NL–G–F^* and WT mice. Therefore, under the experimental conditions we employed, supplementation with *B. breve* MCC1274 did not significantly enhance neurogenesis or cell proliferation in the hippocampus [73,74]. While *B. breve* MCC1274 does not appear to directly stimulate cell proliferation, it may still offer beneficial effects on cognitive function through other mechanisms, such as reducing inflammation, improving synaptic plasticity, and modulating neurotransmitter levels.

We also assessed the impact of *B. breve* MCC1274 supplementation on neuronal health by measuring the protein levels of NeuN, a marker of mature neurons, in brain tissue after four months of supplementation in 17–month–old *App^NL–G–F^* mice. NeuN expression did not show significant alterations in any of the brain regions examined, including the hippocampus and cortex, when comparing the probiotic-supplemented animals to controls [140], indicating that the effects of *B. breve* MCC1274 supplementation on cellular proliferation and neuronal cell loss may be limited. These findings suggest that, despite the potential benefits of *B. breve* MCC1274 on neuroinflammation and the gut–brain axis, the supplementation did not have a direct impact on neuronal viability or maturation as indicated by NeuN levels. This implies that while this probiotic may influence other neurobiological processes, such as synaptic function, they may not induce significant changes in the number of mature neurons or neuronal survival within the timeframe of this study.

### 5.7. The Effects of B. breve MCC1274 Supplementation on Synaptic Protein Levels

Synaptic proteins play a critical role in neurotransmitter release from the presynaptic terminal and the organization of dendritic spines in postsynaptic membranes. The presynaptic proteins are responsible for anchoring synaptic vesicles, regulating their fusion with the cell membrane, and recycling the vesicles after release. These proteins are essential for the formation and maintenance of synapses, which allow for the adaptation and modification of neural connections based on experience, an important process for learning and memory. Additionally, synaptic proteins help maintain the structural stability of the synapse by linking the pre– and postsynaptic components together [141,142]. By influencing the strength of synaptic transmission, synaptic proteins can fine–tune the signal strength between neurons, facilitating complex information processing. Synaptic dysfunction is a key feature of AD and is associated with cognitive decline. Several factors can cause synaptic dysfunction, including Aβ oligomers, mitochondrial dysfunction, and decreased levels of key transmitters such as acetylcholine [143,144,145,146,147]. This dysfunction can be detected early in the progression of AD, even before neuronal degeneration occurs, suggesting that synaptic failure is a significant factor in the disease [148].

In our previous studies, we evaluated the effects of *B. breve* MCC1274 on several presynaptic proteins, such as synaptophysin (SYP), synaptotagmin (SYT), and syntaxin, which are crucial for neurotransmitter release and neuron communication [84,149]. We also examined post-synaptic density 95 (PSD–95), a protein important for receiving and processing signals from other neurons, which supports the formation and stabilization of synaptic connections [150,151] (Table 1). In six-month-old *App^NL–G–F^* mice, the levels of the synaptic proteins SYT and PSD–95 were significantly higher in the supplemented group compared to the vehicle group. This indicates that supplementation with *B. breve* MCC1274 significantly boosted the levels of both pre– and postsynaptic proteins in the hippocampus. Additionally, this supplementation enhanced ERK activation in this area, suggesting that the bacteria could potentially improve memory function by increasing synaptic density through the ERK signaling pathway, potentially leading to increased levels of ADAM10 as well [74]. While the study does not directly explore the molecular pathways involved in synaptic improvement, it suggests that the reduction of Aβ and microglial activation, along with the modulation of inflammation, are central to the observed improvements in synaptic health and memory function.

Furthermore, *B. breve* MCC1274 supplementation resulted in a notable increase in the levels of key pre- and postsynaptic proteins (SYT, SYP, syntaxin, and PSD–95) in the hippocampus of WT mice [73]. In this study, *B. breve* MCC1274 activates the AKT/GSK3β pathway, which reduces tau phosphorylation and lowers PS1 protein levels in the hippocampus. This results in decreased production of soluble AB42, leading to a reduction in neuroinflammation. Ultimately, this may help improve synaptic function. Also, the levels of PSD–95 and SYP were significantly higher in the hippocampus of aged, 17–month–old *App^NL–G–F^* mice compared to those not receiving the supplement, indicating that *B. breve* MCC1274 may rescue or maintain synaptic connections in the aged hippocampus, potentially benefiting cognitive function [76] (Figure 1). *B. breve* MCC1274 administration decreased chronic stress in this study. This decrease in chronic stress may contribute to inhibiting tau hyperphosphorylation and enhancing synaptic density.

Another study demonstrated that oral administration of *B. breve* MCC1274 restores contextual fear extinction in Parkinson’s disease (PD) mice by preventing abnormal changes in hippocampal synaptic plasticity. This effect was linked to normalizing elevated neuropsin levels without activating the cAMP/CREB pathway. In PD mice, the mRNA expression levels of synaptic proteins SYP and PSD-95 were significantly reduced, but treatment with *B. breve* MCC1274 restored these levels to those of the control group [85]. Increased neuropsin expression correlated with changes in synaptic plasticity [152], and *B. breve* MCC1274 maintained the spine density at control levels. Additionally, brain-derived neurotrophic factor (BDNF) levels did not show significant changes in PD mice, indicating that BDNF may not be essential for the effects of *B. breve* MCC1274. Previous research indicated that reduced cAMP/CREB signaling contributes to memory extinction in PD [153], but *B. breve* MCC1274 did not increase hippocampal cAMP levels, suggesting that it utilizes different mechanisms. This implies that *B. breve* MCC1274 may activate other signaling pathways to support synaptic plasticity in PD.

Overall, *B. breve* MCC1274 has the potential to positively impact both pre– and postsynaptic proteins, thereby supporting synaptic function and communication between neurons. These effects are likely to contribute to improved cognitive function and may help protect against synaptic dysfunction and loss in neurodegenerative conditions such as AD. Further research is needed to fully elucidate the molecular mechanisms underlying these effects.

## 6. *B. breve* MCC1274 as a Potential Treatment for Cognitive Behavioral Abnormalities in Neurodegenerative Diseases

AD impacts cognitive function by causing a decline in memory and other cognitive abilities. Addressing these underlying mechanisms can potentially improve the overall quality of life for patients, allowing them to maintain some level of independent functioning for a longer period. Several studies have shown that *B. breve* MCC1274 holds promise in enhancing cognitive function in animal models of AD. In a previous study, mice received intracerebroventricular injections of Aβ 1–42 solution which contained a mixture of monomeric and oligomer forms of Aβ, resulting in a significant reduction in alternation behavior during the Y–maze test compared to control mice, which indicated impaired working memory. Living, heat–killed, or sonicated *B. breve* MCC1274 were orally administered to the mice daily by gavage 1 × 10^9^ organisms in 0.2 mL, starting 2 days before Aβ injection. Daily probiotic administration notably improved this working memory impairment [75]. Importantly, this probiotic did not affect locomotor activity, as there were no significant differences in the total number of entries into the three arms among the tested groups. Furthermore, in the passive avoidance test, Aβ–injected mice displayed significantly lower latency times compared to control mice. This impairment was reversed after the daily administration of *B. breve* MCC1274, indicating its potential to ameliorate memory dysfunction in mice administered Aβ. Researchers attribute this improvement to the upregulation of BDNF, which is essential for learning and memory [154], and the downregulation of hippocampal expression of inflammation and immune–reactive genes [75]. Therefore, the effects of *B. breve* MCC1274 modulation of brain immune response that helps reduce pathology in AD–like mouse models also appear to help prevent cognitive decline in these animals. However, the main limitation of this study is its use of mice, which may not fully replicate human AD pathology or the complexity of cognitive impairments. This study used synthetic Aβ proteins (Aβ25–35 and Aβ1–42), which may not fully represent the complexity of the natural amyloid pathology seen in AD patients, especially regarding the presence of other pathological features like tau tangles.

In our previous study involving six-month-old *App^NL–G–F^* mice, *B. breve* MCC1274 supplementation also prevented memory impairment, as demonstrated by the novel object recognition test. In this study, three-month-old *App^NL–G–F^* mice were assigned randomly into the vehicle and probiotic groups: the vehicle group (*n* = 26) received saline, and the probiotic group (*n* = 26) was supplemented with *B. breve* MCC1274 (1 × 10^9^ cfu/5.56 mg/200 µL saline/mouse) via oral gavage five times/week for four months. During the retention session, the saline–treated group showed no significant difference in the time spent exploring the familiar versus the novel object. In contrast, the probiotic-treated group spent significantly more time exploring the novel object [74]. The discrimination index was also significantly higher in the probiotic group compared to the vehicle group, suggesting that *B. breve* MCC1274 can effectively mitigate memory impairment in *App^NL–G–F^* mice by reducing Aβ plaque deposition in the hippocampus and modulating inflammatory responses in the brain [74]. However, the main limitation of this study is the limited exploration of mechanisms; although the study mentions the reduction of Aβ and microglial activation, the precise mechanisms by which *B. breve* MCC1274 affects these processes, such as gut–brain axis interactions or immune modulation, are not fully explored, which limits understanding of how the probiotic exerts its effects.

Additionally, in another study involving 17–month-old *App^NL–G–F^* mice, the administration of *B. breve* MCC1274 significantly improved cognitive impairments [76]. The transgenic mice used in the experiments were aged 13 months. The mice were administered *B. breve* MCC1274 (1 × 10^9^ cfu/mouse/day) via oral gavage five times per week for four months (*n* = 17). Another group of mice that received saline was considered the control group (*n* = 16). The cognitive dysfunction, characterized by difficulty distinguishing between familiar and novel objects in the novel object recognition test, was evident in the saline-treated group but not in those treated with the probiotic. Mice in the probiotic group spent more time exploring the novel object, demonstrating their ability to recognize and differentiate it from the familiar one [76]. The administration of *B. breve* MCC1274 might improve conditions related to AD in 17–month–old *App^NL–G–F^* mice, in part by reducing chronic stress through the inhibition of tau hyperphosphorylation. These studies support the beneficial effect of *B. breve* MCC1274 in alleviating cognitive dysfunction in AD model mice. However, the main limitation of this study is behavior testing; they only used the NOR test for cognition. They have not conducted comprehensive or diverse behavioral tests to assess all aspects of cognitive function and memory. In addition to no long-term follow–up to assess whether the improvements in memory are sustained over time or if any adverse effects emerge after prolonged probiotic use.

Several studies have explored the effects of *B. breve* MCC1274 supplementation on cognitive function in elderly individuals with MCI (Table 2). One study utilized the Repeatable Battery for the Assessment of Neuropsychological Status (RBANS), a diagnostic tool for MCI, along with the Mini–Mental State Examination (MMSE) to evaluate the effect of this probiotic on MCI subjects over a 12–week supplementation period. Both the *B. breve* MCC1274 and placebo groups showed significant improvements in RBANS and MMSE scores, with no significant difference between the groups. In the low-score subgroup, *B. breve* MCC1274 supplementation significantly improved scores in immediate memory and delayed memory, suggesting its potential effectiveness in treating MCI and early stages of dementia. However, no improvement was seen in the high–score subgroup, likely due to their already high baseline scores. Interestingly, the placebo group also showed a greater increase in MMSE scores, indicating a need for further studies on probiotics in individuals with normal cognition [155]. SPSS version 19 (IBM, Tokyo, Japan) was used for statistical analyses in this study.

In anothor study, participants diagnosed with MCI took *B. breve* MCC1274 capsules for 24 weeks, and their cognitive function was assessed using the MMSE and the Digit Symbol Substitution Test (DSST). The results showed significant improvement in MMSE scores after 16 weeks of supplementation, and by the end of the study, 13 out of 19 participants demonstrated cognitive normality, indicating enhanced cognitive function and a reduced risk of dementia. However, no significant change was observed in DSST scores, likely due to physical limitations among the older participants [79]. All statistical analyses in this study were performed using SAS version 9.4 (SAS Institute, Inc., Cary, NC, USA). In a recent study, supplementation with *B. breve* MCC1274 over 16 weeks led to significant improvements in cognitive function, as measured by RBANS scores, in subjects with MCI compared to those receiving a placebo. Notable improvements were found across several RBANS domains, including immediate memory, visuospatial/constructional skills, and delayed memory, although no improvements were observed in language and attention scores. These findings suggest that *B. breve* MCC1274 positively impacts memory, especially in individuals with suspected MCI, and may be more effective than other dietary supplements for this population. Previous studies have indicated reductions in medial temporal lobe (MTL) volumes and hippocampal shrinkage in MCI subjects [156,157]. Therefore, the observed improvements in immediate and delayed memory following *B. breve* MCC1274 supplementation may reflect positive changes in the hippocampus, a crucial brain region for memory. Additionally, preclinical studies in mice have shown that *B. breve* MCC1274 improves memory and reduces inflammation in the hippocampus, suggesting potential similar effects in humans [81]. Statistical analysis was performed using SPSS software version 26 (IBM, Tokyo, Japan) in this study.

Another supporting study revealed that lower serum levels of hemoglobin A1c correlate with improved cognitive function in MCI subjects, as reflected in RBANS total scores, possibly indicating a mechanism related to inflammation reduction. Furthermore, a reduction in albumin levels has been associated with an increased risk of MCI and AD, implying that *B. breve* may help slow the progression of MCI symptoms by preventing declines in albumin levels [62]. A stratified analysis based on baseline HbA1c with a median value and statistical analysis of the safety biomarkers was performed with Student’s *t*-test in this study. In yet another study, MCI subjects who consumed *B. breve* MCC1274 showed cognitive improvements in certain subscales of the Japanese version of the Alzheimer’s Disease Assessment Scale (ADAS–Jcog) and the MMSE, although total scores did not reveal significant changes. Probiotic supplementation appeared to slow the progression of brain atrophy in these patients, as measured by the Voxel–based Specific Regional Analysis System for Alzheimer’s Disease (VSRAD) brain MRI, with no changes in gut microbiota composition. Participants with more severe cognitive impairment exhibited significant improvements in the “orientation” subdomains of both the ADAS–Jcog and the MMSE [80]. Statistical analysis was performed using SAS software version 9.4 (SAS Institute Inc., Cary, NC, USA) for neuropsychological data or R software ver. 3.6.0 for VSRAD data in this study. As discussed, *B. breve* MCC1274 may have cognitive benefits comparable to or exceeding some AD treatments. It can potentially prevent cognitive decline in both AD model mice and MCI subjects while offering a safer side effect profile. However, other treatments like cholinesterase inhibitors and memantine mainly provide symptomatic relief that can demonstrably improve cognitive function in individuals experiencing dementia, particularly in the early to moderate stages of AD, with several side effects, although the improvement may be modest and not completely halt disease progression, whereas *B. breve* MCC1274 may address underlying mechanisms and potentially slow disease progression. In addition, other probiotics such as *Lactobacillus* species and *Bifidobacterium bifidum* have also shown cognitive benefits, but their specific mechanisms and efficacy may vary. Overall, *B. breve* MCC1274 supplementation showed promise in improving cognitive function in patients with MCI, as evidenced by several studies.

Moreover, *B. breve* MCC1274 may help reduce symptoms associated with other mental health conditions; it has been indicated that supplementation with *B. breve* MCC1274 can lead to significant improvements in anxiety and depression scores in patients with schizophrenia, particularly when combined with monitoring of baseline gut microbiota composition to identify potential responders to this probiotic treatment [90]. All statistical analyses were performed using R version 4.0.3 (R Core Team, Vienna, Austria), ggplot2, and the dplyr packages in this study. It has been suggested that elevated lipid and energy metabolism at baseline may be linked to the effect of probiotics on anxiety and depressive symptoms. The end-products of lipid and energy metabolism produced by gut microbiota could help maintain a healthy gut environment and influence the symptoms related to systemic inflammation [158]. Higher relative abundances of functional lipid and energy metabolism pathways were observed in responders versus non-responders to probiotics, suggesting that the effects of *B. breve* MCC1274 may depend on sufficient metabolic function.

Gut microbiota produces SCFAs from indigestible foods, which are crucial for preventing obesity and maintaining gut health, thereby potentially affecting anxiety and depression. SCFAs also serve as an energy source for intestinal cells and promote beneficial bacteria while inhibiting harmful ones. As mentioned earlier, *B. breve* MCC1274 has been shown to enhance lipid metabolism, increase butyrate production, activate regulatory T cells, and reduce systemic inflammation. Overall, these findings indicate that lipid metabolism may play a significant role in the anti-inflammatory effects of *B. breve* MCC1274 on anxiety and depressive symptoms. Another study confirmed the beneficial role of *B. breve* MCC1274 in alleviating anxiety and depressive symptoms. This probiotic showed promise in improving anxiety and depression in schizophrenia patients, particularly those with low dairy intake and high Parabacteroides levels [82]. The improvements seem to stem from enhancing gut barrier function rather than changes in the gut microbiome itself, as indicated by increased IL–22 and TRANCE levels in responders, which are crucial for maintaining this barrier. All analyses were performed using R.3.3.3 software (R Development Core Team, 2016) and the following R packages: stats for multiple comparisons and ggplot2 for graphics in this study. Further research should explore these effects in other psychiatric conditions and assess dietary habits to personalize probiotic treatments. As affective disorders such as anxiety and depression are also observed in prodromal AD stages such as MCI, studies from neuropsychiatric diseases may also inform additional mechanisms of AD therapy.

## 7. Comparison of *B. breve* MCC1274 with Other Probiotic Strains in AD Progression and Brain Health

The potential of probiotics to influence the progression of AD and support brain health is an exciting area of research, particularly in the context of the gut–brain axis. While *B. breve* MCC1274 has shown promising results in preclinical models and early-stage human studies, it is crucial to evaluate how this strain compares with other probiotics in terms of its ability to affect AD progression and cognitive function. For instance, as shown in Table 4, other strains such as *Lactobacillus rhamnosus* GG and *Bifidobacterium longum* 1714 also exhibit similar effects on brain health. These probiotics have been found to enhance cognitive function and reduce anxiety-like behaviors, potentially through their action on the CNS and their modulation of the gut microbiome [159,160,161]. Some evidence suggests that *Lactobacillus rhamnosus* GG can influence GABA receptors in the brain, which could have a beneficial effect on anxiety and stress-related disorders [162].

As discussed above, *B. breve* MCC1274 has shown significant potential to reduce neuroinflammation by promoting the production of anti-inflammatory mediators and reducing the pro-inflammatory cytokines and regulating microglial activation. Other probiotics, such as *Lactobacillus rhamnosus* GG have also demonstrated neuroprotective effects by regulating the immune response and neuroinflammation in the brain through increasing IL-10 and regulatory T cells and decreasing pro-inflammatory cytokines [161,163]. Moreover, *Bifidobacterium infantis* 35624 has shown immunomodulatory effects by reducing plasma IL–6 and TNF–α levels [164]. while *Bifidobacterium longum* 35624 did not affect immune function [165]. However, the combination of *Lactobacillus helveticus* R0052, *Bifidobacterium longum* subsp. *infantis* R0033, and *Bifidobacterium bifidum* R0071 has demonstrated promising effects in reducing neuroinflammation and enhancing cognitive function in preclinical studies [166].

In addition, similar to the data showing that *B. breve* MCC1274 may reduce toxic Aβ levels, other probiotic strains such as *Bifidobacterium bifidum* BGN4 and *Bifidobacterium longum* BORI were shown to effectively suppress amyloidosis and apoptotic processes in the 5xFAD mouse model of AD [167]. Additionally, *Lactobacillus plantarum* PS12 has been shown to prevent Aβ deposition in a 3×Tg–AD mouse model through the inhibition of BACE1 and GSK–3β [168].

Overall, when comparing *B. breve* MCC1274 with other probiotic strains, it is evident that it possesses unique potential in influencing AD progression, Aβ accumulation, neuroinflammation, and overall brain health, mainly through its effects on the gut–brain axis. However, other strains particularly *Lactobacillus rhamnosus* GG and *Bifidobacterium longum* 1714, have shown similar effects in preclinical studies and may provide comprehensive benefits for cognitive function and neuroinflammation. Translating preclinical findings into effective clinical treatments for AD, especially using probiotics like *B. breve* MCC1274, *Lactobacillus rhamnosus* GG, and *Bifidobacterium longum* 1714, is fraught with challenges. These include variability in individual responses, the complexity of AD pathology, the need for long–term studies to assess efficacy and safety, the difficulty of standardizing probiotic interventions, and regulatory hurdles. Overcoming these challenges will require rigorous clinical research, innovative trial designs, and a nuanced understanding of the gut–brain axis in the context of AD. More research, particularly in human clinical trials, is needed to fully understand the comparative efficacy of these strains in AD and other neurodegenerative diseases.

While *B. breve* MCC1274 has demonstrated its effectiveness in improving cognition in subjects with MCI in several studies, other probiotics such as *Lactobacillus fermentum*, *Lactobacillus plantarum*, *Bifidobacterium lactis*, *Lactobacillus acidophilus*, *Bifidobacterium bifidum*, *Bifidobacterium longum*, *Lactobacillus reuteri*, *Lactobacillus GG AT* strain 53103, and yogurt drinks containing *Lactobacillus casei*, *Bifidobacterium infantis*, and *Lactobacillus rhamnosus* do not appear to influence cognitive development in humans [169,170,171,172,173]. In contrast, *Lactobacillus plantarum* p8 has been shown to significantly enhance social–emotional cognition in women and improve recognition memory in men following probiotic intervention compared to a placebo [174]. Additionally, multispecies probiotics containing *Lactobacillus rhamnosus* and *Bifidobacterium lactis* have been associated with improved cognitive function (mean difference of 1.90, 95% CI 1.09 to 2.70, *p* < 0.005), enhanced memory (mean difference of 4.60, 95% CI 2.91 to 6.29, *p* < 0.005) as measured by the MMSE and digit tasks, and a reduction in depressive symptoms (mean difference of 4.09, 95% CI 1.70 to 6.48, *p* < 0.005) according to the Beck Depression Inventory [175].

## 8. Future Directions and Conclusions

Research on the gut–brain axis and its role in neurodegenerative diseases, particularly AD, is rapidly expanding, and in this review, we have shown how *B. breve* MCC1274 specifically may modulate this axis to reduce AD pathophysiological pathways and positively impact memory and cognitive behavior. However, more robust clinical evidence and several key areas are required. The key areas for future research include investigating the effectiveness, safety, and optimal usage of *B. breve* MCC1274 in AD patients and those at risk; exploring the underlying mechanisms by which *B. breve* MCC1274 influences brain health and AD pathophysiology; examining combinations with pharmacological agents, dietary interventions, and other probiotics to enhance the benefits of *B. breve* MCC1274; and developing tailored interventions based on individual microbiome and genetic profiles to maximize therapeutic outcomes. Through these research avenues, *B. breve* MCC1274 may become a crucial component of future strategies for preventing and managing AD, offering a novel and targeted approach to this complex neurodegenerative disorder.

In conclusion, *B. breve* MCC1274 shows considerable promise as a potential therapeutic agent in the prevention and management of AD. Emerging evidence suggests that this probiotic strain may exert protective effects through various mechanisms, including modulation of the gut–brain axis, reduction of neuroinflammation, improvement of cognitive function, and promotion of neuroprotective compounds in animal models of AD. The ability of *B. breve* MCC1274 to positively influence cognitive function, mood, and overall brain health holds significant therapeutic potential, especially for patients in the early stages of AD or those at risk. Overall, *B. breve* MCC1274 represents a promising new frontier in Alzheimer’s research, with the potential to contribute to innovative, microbiome-based treatment strategies. Continued research and clinical trials are crucial to unlocking its full potential as part of a comprehensive approach to AD prevention and management.

## Figures and Tables

**Table 1:** The different mechanisms and effects associated with *B. breve* MCC1274 supplementation in experimental animals.

Effect	Mouse Model	Description	Findings/Mechanism	Reference
Neuroinflammation Modulation	Aβ–injected mice	Suppressed inflammation and immune–reactive genes.	DEGs observed in Aβ–injected mice returned to normal expression levels in the hippocampus.	[71]
	6-month–old *App^NL–G–F^* mice	Shift in microglial phenotype from M1 to M2 (reduced inflammation around Aβ plaques).	Decreased IL–1β, TNF–α, Iba1 protein, and Iba1^+^ cells and increased anti–inflammatory cytokines like TGF–β1.	[70]
	WT mice	Attenuated microglial activation	Reduced protein and the number of Iba1^+^ cells by Akt/GSK–3β pathway.	[69]
Oxidative Stress & Chronic Stress Response	17–month–old *App^NL–G–F^* mice	Reduction of chronic stress markers	Decreased protein levels of p–JNK, p–ERK1/2, and HSP90	[72]
	In vitro cell culture	Reduction of oxidative stress	Reduced perilipin 4 and lipid droplet accumulation in neurons	[73]
Amyloid Pathology Modulation	Aβ–injected mice	Lowering Aβ toxicity	Production of beneficial SCFAs such as acetate	[71]
	6–month–old *App^NL–G–F^* mice	Reduction in Aβ deposition	Increased ADAM10 enzyme activity	[70]
	WT mice	Reduction in soluble Aβ42	Decreased PS1 enzyme activity	[69]
	17–month–old *App^NL–G–F^* mice	No effect on amyloid pathology	No significant reduction in Aβ levels	[72]
Tau Pathology Modulation	6-month–old *App^NL–G–F^*	No effect on tau phosphorylation	Phosphorylated tau levels at Ser202/Thr205 and Thr231 had no change	[70]
	WT and 17–month–old *App^NL–G–F^* mice	Reduction in tau phosphorylation	Reduced phosphorylated tau levels at Thr231 and Ser202/Thr205 (WT) as well as Thr231 and Ser396/Ser404 (*App^NL–G–F^*)	[69,72]
Gut Microbiota	Aβ-injected and *App^NL–G–F^* mice	No change in the overall gut microbiota	No increase or decrease in the abundance of other bacterial species	[70,71]
	Female Wistar rats	Did not restore microbial diversity after antibiotic treatments	Increase the relative abundance of *Actinobacteria* and the total counts of *B. breve*	[83]
Blood–Brain Barrier Integrity	Aβ-injected, 6–month–old *App^NL–G–F^* and WT mice	Potential protection of BBB from neuroinflammation	Reduced neuroinflammation and Aβ accumulation which can protect the BBB	[69,71]
Synaptic Protein Levels	6–month–old *App^NL–G–F^*	Increase in synaptic protein expression	Increase in SYT and PSD–95 protein levels	[70]
	WT mice	Increase in synaptic protein expression	Increase in SYT, SYP, syntaxin, and PSD–95 protein levels	[69]
	17–month–old *App^NL–G–F^*	Increase in synaptic protein expression	Increase in SYP and PSD–95 protein levels	[72]
	PD mice	Improved synaptic plasticity	mRNA expression levels of SYP and PSD–95 were restored	[84]
Behavioral Improvements	Aβ–injected mice	Improvement in cognitive function and behavior	Improved working memory (Y–maze) and memory retention (passive avoidance test)	[71]
	6–and 17–month–old *App^NL–G–F^*	Ameliorated cognitive decline and improved memory	Increased exploration of novel object and discrimination index in NOR test.	[70,72]
Cellular Proliferation & Neuronal Cell Loss	6–month–old *App^NL–G–F^* and WT mice	No effect on neurogenesis	No significant increase in cellular proliferation (BrdU–positive cells)	[69,70]
	17–month–old *App^NL–G–F^*	No effect on neuronal loss	No significant change in mature neuronal marker NeuN levels	Not published

**Table 2:** Summary of human studies on the effects of B. breve MCC1274 on various neurodegenerative and mental health conditions, with a focus on cognitive function, anxiety, and depression.

Disease and Duration of Supplementation	Effects	Test(s) Used for Assessment	Effect on Inflammation	Effect on Gut Microbiota	Reference
Mild Cognitive Impairment (12 weeks)	Significant improvement in immediate and delayed memory in low-score subgroup.	BRANS and MMSE	Reduction in systemic inflammation, likely via SCFAs and anti–inflammatory metabolites.	No significant changes in gut microbiota composition.	[85]
Mild Cognitive Impairment (24 weeks)	Significant improvement in MMSE scores after 16 weeks, with some reaching cognitive normality.	MMSE and DSST	Potential reduction in inflammation, as observed in improved cognitive scores.	No significant changes in gut microbiota composition.	[75]
Mild Cognitive Impairment (16 weeks)	Improvement in memory, especially in RBANS domains such as immediate and delayed memory.	RBANS	Potential reduction in inflammation in hippocampus, linked to cognitive improvement.	No significant changes in gut microbiota composition.	[77]
Mild Cognitive Impairment (16 weeks)	Improvement in cognitive function, as reflected in RBANS total scores	RBANS	Lower serum levels of hemoglobin A1c correlate to reducing inflammation	Not evaluated	[58]
Mild Cognitive Impairment (12 weeks)	Improvement in the orientation subdomains of ADAS–Jcog and MMSE; slower progression of brain atrophy.	ADAS–Jcog, MMSE, and VSRAD brain MRI	Slower progression of brain atrophy, indicating reduced neuroinflammation	No significant changes in gut microbiota composition.	[76]
Schizophrenia (4 weeks)	Significant improvement in anxiety and depression symptoms.	Anxiety and depression scores	Significant reduction in anxiety/depression linked to reduced inflammation via improved gut barrier function by increased IL–22 and TRANCE	No changes in gut microbiota composition but related to gut barrier function	[78]
Schizophrenia (4 weeks)	Significant improvement in anxiety and depressive symptoms, linked to lipid metabolism.	Anxiety and depression scores	Enhanced lipid metabolism linked to reduced inflammation, improving anxiety/depression symptoms	No changes in gut microbiota composition, but related to metabolic effects	[86]

**Table 3:** Summary of the effects of *B. breve* MCC1274 on various metabolites and immunomodulatory properties.

Metabolite	Model/System	Influence	References
Genistein	*App^NL–G–F^* and WT mice	Increases plasma levels; potential anti–inflammatory and antioxidative activities, reduces inflammation oxidative stress in the brain	[74]
Acetate	Aβ-injected and *App^NL–G–F^* mice, and MCI	Alleviate neuroinflammation and can decrease amyloid pathology by lowering the levels of both soluble and insoluble fractions of hippocampal Aβ1–42	[71,74,77]
5–methoxyindoleacetic acid	*App^NL–G–F^* mice	Exhibits antioxidative activity; reduces oxidative stress not only in the brain but throughout the body, providing broader protective effects	[74]
Indole–3–lactic acid	*App^NL–G–F^* mice and MCI	Impact intestinal barrier function and immune regulation, rather than causing substantial changes to bacterial diversity	[74]
Daidzein	Wistar rats (antibiotic pre-treated)	Reduce inflammation and oxidative stress by increasing bioavailability by hydrolyzing daidzin into a more absorbable form	[83]
SCFAs	*App^NL–G–F^* mice, MCI, and Schizophrenia	Promotes gut health, modulates inflammation and microglial activity	[74,76,86]
Brain-derived neurotrophic factor	Aβ–injected mice	Ameliorate memory dysfunction in mice administered Aβ	[71]
Lipid Droplet Metabolites	SH-SY5Y cells	Reduces lipid droplet formation and expression of perilipin 4 in neurons	[73]
IL–1β, TNF–α	*App^NL–G–F^* mice	Reducing pro–inflammatory cytokines like IL-1β and TNF–α directly contributes to reducing inflammation in the body	[70]
TGF–β1	*App^NL–G–F^* mice	Increased anti–inflammatory cytokines like TGF–β1 help to dampen excessive immune responses and reduce inflammation in the body	[70]
Lipopolysaccharides	MCI	Elevated gut inflammation levels, but it reduced with *B. breve* supplementation	[77]

**Table 4:** Comparison *B. breve* MCC1274 with other probiotic strains based on their effects on AD progression, Aβ production, neuroinflammation, and brain health.

Bacterial Strain	Effect on AD Progression	Effect on Aβ Production	Effect on Neuroinflammation	Effect on Brain Health	Reference
*B. breve* MCC1274	Potential to reduce AD progression by modulating gut–brain axis and improving cognitive function	Reduce Aβ levels in AD model mice, possibly by altering APP processing enzymes or via effects on neuroinflammation	Reduces neuroinflammation, likely through modulation of microglial activation and inflammatory cytokines	Improves cognitive function, reduces anxiety and depression, and enhances memory	[69,70,71,72]
*Lactobacillus rhamnosus* GG	Enhances cognitive function and reduces anxiety–like behaviors, potentially benefiting AD patients	Some evidence suggests a potential effect on GABA receptors but no direct data on Aβ reduction in AD.	Regulates immune response, increases IL–10 and regulatory T cells, and decreases pro-inflammatory cytokines.	Improves anxiety, stress–related behaviors, and cognitive function.	[155,157,158,159]
*Bifidobacterium longum* 1714	Potential cognitive benefits with improvements in mental health.	No direct evidence on Aβ reduction but may influence gut–brain communication	Modulates neuroinflammation by influencing cytokine production and reducing pro-inflammatory signals.	Enhances cognitive function, reduces anxiety, and boosts mental well-being.	[156,161]
*Bifidobacterium infantis* 35624	May have neuroprotective effects, particularly in improving mood and cognitive function.	Some studies suggest immunomodulatory effects that could indirectly affect Aβ	Reduces plasma IL–6 and TNF–α levels, suggesting a reduction in neuroinflammation.	Improves mood and cognitive function by modulating gut–brain axis.	[160]
*Bifidobacterium bifidum* BGN4	Reduces amyloidosis and apoptotic processes in AD model mice.	Effectively suppresses amyloid deposition in AD model mice.	Potential to reduce neuroinflammation and related damage in the brain.	Shows promise in improving brain health in preclinical AD models.	[163]
*Bifidobacterium longum* BORI	Suppresses amyloidosis and mitigates neuronal damage in AD models	Suppresses Aβ accumulation in AD mouse models	Reduces neuroinflammation, likely via modulation of the gut–brain axis.	Shows improvements in cognitive health in AD models	[163]
*Lactobacillus plantarum* PS12	Potential neuroprotective effects in AD models	Inhibits Aβ deposition in 3×Tg–AD mouse models by inhibiting BACE1 and GSK3β	Modulates neuroinflammation by affecting cytokine and immune responses	Enhances brain health and reduces cognitive decline in AD models	[164]
*Lactobacillus helveticus* R0052, *Bifidobacterium longum* R0033, *Bifidobacterium bifidum* R0071	Shows promising preclinical effects in improving cognition and reducing AD progression	No direct evidence on Aβ reduction, but likely modulates brain health through gut-brain interactions	Reduces neuroinflammation in AD models by improving gut microbiome balance	Improves cognitive function and reduces AD–like pathology in preclinical models	[162]
